# Risk factors for hamstring strain injury in male college American football players -a preliminary prospective cohort study-

**DOI:** 10.1186/s12891-023-06565-w

**Published:** 2023-06-02

**Authors:** Yuri Mizutani, Shuji Taketomi, Kohei Kawaguchi, Seira Takei, Ryota Yamagami, Kenichi Kono, Tomofumi Kage, Shin Sameshima, Hiroshi Inui, Sayaka Fujiwara, Sakae Tanaka, Toru Ogata

**Affiliations:** 1 UTokyo Sports Science Institute (UTSSI), Komaba I Campus, 3-8-1, Komaba, Meguro-Ku, 3rd Floor, Bldg.9, Tokyo, 153-8902 Japan; 2 Department of Orthopaedic Surgery Faculty of Medicine, The University of Tokyo, 7-3-1, Hongo, Bunkyo-Ku, Tokyo, 113-8655 Japan; 3 Department of Rehabilitation Medicine, The University of Tokyo, 7-3-1, Hongo, Bunkyo-Ku, Tokyo, 113-8655 Japan

**Keywords:** Hamstring strain injury, Hamstring flexibility, Hamstring to quadriceps strength ratio, General joint laxity, Risk factor, College American football player, Injury prevention

## Abstract

**Background:**

Given the frequency of hamstring strain injuries (HSI) among male college American football players, several studies have attempted to determine whether certain risk factors can predict their occurrence. However, no consensus on modifiable risk factors for HSIs in male college American football players has yet been reached to prevent these injuries. This study aimed to clarify risk factors for HSI prospectively in college male American football players.

**Methods:**

A total of 78 male college American football players, whose positions were limited to skill positions, were medically assessed for potential risk factors of HSI. The preseason medical assessment included anthropometric measurements, joint laxity and flexibility, muscle flexibility, muscle strength, and balance ability.

**Results:**

HSI occurred in a total of 25 thighs from 25 players (32.1%). Injured players had significantly lower hamstring flexibility (*p* = 0.02) and hamstring to quadriceps strength ratio (H/Q) (*p* = 0.047) compared to uninjured players. Additionally, injured players had significantly lower general joint laxity scores, especially for the total (*p* = 0.04), hip (*p* = 0.007), and elbow (*p* = 0.04) scores, compared to uninjured players.

**Conclusions:**

Lower hamstring flexibility, lower hamstring to quadriceps strength ratio, and lower general joint laxity score were identified as risk factors for HSI in male college American football players placed in skill positions. The muscle flexibility and H/Q ratio could be useful in preventing HSI in such players.

## Background

American football, a complex sport that requires athletes to perform different skills and activities depending on the player’s position, is quite popular among male college students. Moreover, it is a high-impact collision sport that can promote injuries in both contact and noncontact situations [1], leading it to be recognized as one of the highest-risk sports for injury. Studies have shown that American football has a game injury rate of 35.9 injuries per 1000 Athlete-Exposures (AE), whereas men’s soccer and baseball have injury rates of only 18.8 and 5.8 injuries per 1000 AE [2]. Shankar et al. reported that over 500,000 American football-related injuries occurred in the United States during the 2005–2006 season at all game levels [3].

Hamstring strain injury (HSI), one of the most common and often serious musculoskeletal injuries observed in American football [4, 5], requires lengthy rehabilitation and places the athlete at a distinct risk for reinjury, with estimates showing that approximately one-third of the HSI sustained by collegiate student athletes were recurrent [6, 7]. At least two types of acute HSI exist. The first and most common injury type occurs during high-speed running, whereas the other occurs during other movements, such as high kicking, sliding tackles, and sagittal splitting, which cause extensive hamstring lengthening [8]. In American football players, HSIs have mostly been caused by noncontact incidents and occur while running [1, 6]. The incidence of HSI is high in skill positions in American football [1, 3, 4, 6, 7, 9, 10]. Therefore, research should focus on identifying risk factors for HSI in college American football players in skill positions to prevent such injuries from occurring. Several risk factors have been proposed to predict the occurrence of HSI. Notably, previous injuries have been proposed as the greatest single contributor to future HSI not only in American football [7] but also in other football sports, such as soccer [11], Australian football [9, 11–15] and rugby [16]. Extrinsic risk factors, such as weather [9], situations (practice or game) [1], and seasons [1], have also been investigated as potential contributors. However, potential intrinsic risk factors, including muscle strength [7, 16–24], muscle flexibility [22, 25–27], and lower limb range of motion [22, 28], have been considered more clinically relevant given that they are modifiable risk factors. Nonetheless, their significance still remains controversial. It may be caused by the differences in the measurement methods or in the sport; therefore, we limited the study participants to American football players whose positions are skill positions.

Furthermore, only a handful of studies have investigated risk factors for HSIs among American football players, warranting the need for prospective cohort studies on multiple possible factors involving the lower extremities of American football players, especially in skill positions. This study aimed to prospectively identify risk factors for HSI involving the lower extremities of young male American football players in skill positions.

## Methods

### Participants

This study included male college American football players on one team whose positions were limited to skill positions, such as quarterback, running back, wide receiver, linebacker, and defensive back, because skill positions are one of the risk factors for HSI in American football [1, 3, 4, 6, 7, 9, 10] and there are differences in physique, body composition, and muscle strength between the skill positions and linemen, such as defense line, offense line, and tight end. Freshmen were also excluded given that they received freshman training, which differed from the team’s training consisting of American football-specific exercises. The athletes with lower limb injuries that occurred within 12 weeks were excluded from this study. Finally, 78 players were enrolled. In particular, the history of HSI is recorded as an evaluation item for this study. Data obtained from preseason participation physical screenings over 2 years (2019–2020) were prospectively analyzed. Players’ injury registration continued from study onset until the final competition of each season. Consequently, only one set of measurement data was used for each player within this period. The latest data were used for players who participated in the medical assessment twice. This study is part of a prospective study on predictors of sports injuries, namely the University of Tokyo Sports Science Initiative (UTSSI) Sport Injury Prevention Project [29, 30], which includes athletes from other sports such as soccer. This sports injury prevention project was approved by the Institutional Review Board of the University of Tokyo. The participants provided comprehensive written consent to participate in the study, including publication.

### Measurements

This study used data obtained from six types of measurements: anthropometric measurements, general joint laxity tests, muscle flexibility tests, lower limb range of motion, muscle strength tests, and balance tests. Because this study is a part of the UTSSI injury prevention project and the purpose of the project is to reveal the risk factors not only for HSI but also for other sport injuries, such as ACL injuries, ankle sprains, and stress fractures, we chose them to measure at the medical assessment, as they were considered to be the risk factors for such injuries. All measurements were performed by skilled sports physicians, who were assigned to each test in the preseason medical assessment.

#### Anthropometric measurements

Body weight and body height were measured for each player, after which their body mass index (BMI) was calculated. Body composition was measured using InBody 270 (Biospace Co., Ltd., Seoul, Korea), a multifrequency impedance analyzer that can determine each player’s skeletal muscle mass, total body minerals, body fat mass, and percent body fat.

### Muscle flexibility tests

Muscle flexibility tests were performed bilaterally on the iliopsoas, quadriceps femoris, hamstring, gastrocnemius, and soleus muscles. Reports have found that this flexibility testing had excellent intra-rater reliability for all muscle flexibility measures (intraclass correlation coefficients = 0.89–0.96) [31].

#### Iliopsoas

**Fig. 1:**
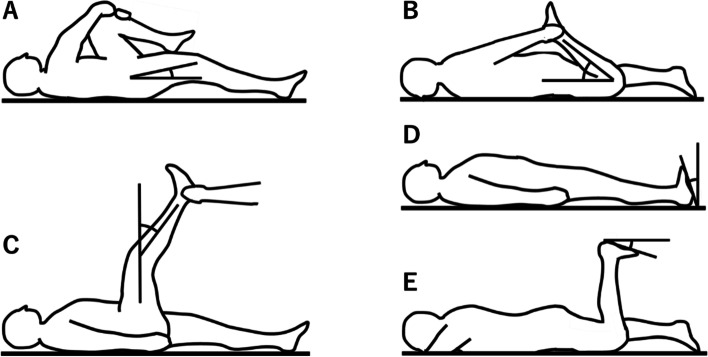
Muscle flexibility tests were performed bilaterally on the iliopsoas (**A**), quadriceps (**B**), hamstring (**C**), gastrocnemius (**D**), and soleus muscles (**E**)

Iliopsoas muscle measurements were performed by obtaining the angle of the hip joint during maximal passive bending of the opposite hip joint with the participants’ hands in the supine position (Fig. 1A).

#### Quadriceps

Participants grasped their lower leg just proximal to the ankle and pulled it toward the buttocks to measure quadriceps flexibility. Quadriceps muscle measurements were performed by bending the angle of the knee joint while positioned prone. The examiner verbally reminded the participants not to lift their buttocks by tensing their muscles during the measurement (Fig. 1B).

#### Hamstrings

Hamstring muscle flexibility was performed with the hip at 90° of flexion while positioned supine. The examiner held the participant’s heel, after which the angle between the vertical line to the floor and the long axis of the tibia after maximally extending the knee joint was measured as hamstring muscle flexibility (Fig. 1C).

#### Gastrocnemius

The ankle joint active dorsiflexion angle was measured at maximal dorsiflexion while positioned supine, with the knee extended and maintained in a neutral position relative to the varus-valgus angle of the ankle, to measure gastrocnemius muscle flexibility (Fig. 1D).

#### Soleus

The ankle joint active dorsiflexion angle was measured at maximal dorsiflexion while positioned prone with the knee at 90° of flexion to measure soleus muscle flexibility (Fig. 1E).

#### Joint Range of motion

##### Internal rotation of hip

Passive hip internal rotation angle was measured while positioned prone with their hips in a neutral position and their knees flexed to 90 degrees.

##### Knee extension

Knee hyperextension angle was measured while standing with the involvement of the quadriceps.

##### Ankle dorsiflexion

The weight-bearing maximum ankle dorsiflexion angle with knee flexion was also measured.

#### Muscle strength tests

##### Isometric knee extension and flexion

A Biodex Multi-Joint System 3 (Biodex Medical Systems Inc., Shirley, NY, USA) was used to measure knee flexion and extension muscle strength. The player performed a 5-min warm-up routine involving cycling on a stationary exercise bicycle before undergoing measurements. The order at which each side was measured was randomized. The test was composed of isometric contraction with knee flexion and extension at 70°. The highest peak torque value was recorded. Strength measures were normalized to BW. The hamstring–quadriceps (H/Q) ratio was also calculated. The average of the left and right sides was used for further analysis.

##### Isometric hip abduction

Hip abductor strength was measured isometrically using a handheld dynamometer (μTAS F-1; Anima Industry Inc., Tokyo, Japan). All participants laid supine with their hips in a neutral position beside the wall, with both knees extended and their arms crossed over their chest to allow for a standardized testing procedure. Participants were instructed to abduct the leg as much as possible over 5 s, with 1 min of rest between contractions. Consequently, the peak force was recorded for further analysis. The dynamometer was placed on the lateral epicondyle of the femur, after which the distance between the lateral epicondyle and hip center was measured. Isometric hip abductor strength was assessed using a handheld dynamometer with good to excellent intra- and intertester reliability. Furthermore, the highest peak torque value was recorded. Strength measures were normalized to BW. The average of the left and right sides was used for further analysis.

#### General joint laxity

The general joint laxity of each player was assessed using the methods of the University of Tokyo described by Watanabe et al. [32]. These general joint laxity tests consisted of wrist and thumb to forearm opposition, elbow hyperextension ≥ 15°, shoulder hyperrotation, hip hyperexternal rotation ≥ 90° in the standing position, knee hyperextension ≥ 10°, ankle hyperdorsiflexion ≥ 45° with knee flexion, and palms to floor with knees fully extended. Positive shoulder hyperrotation was defined as a condition wherein participants could clasp their hands from both the cranial and caudal sections of their back. Positive hip hyperexternal rotation was defined a condition wherein participants could maintain their hips at 90° of external rotation with both their lower legs in a neutral position. All tests except trunk flexion and hip external rotation were performed bilaterally. A point value of 0.5 was awarded each time a player surpassed the designated laxity measure at each of the five joints tested (both wrists, elbows, shoulders, knees, and ankles), whereas 1 point was awarded for surpassing the designated measure for two tests (trunk and hip). The total points were summed, with a maximum score of 7.

#### Balance tests

##### Double- and single-leg balance

A 1-m Footscan pressure plate (RSscan International, Flanders, Belgium), with 8,192 resistive sensors and 5.08 × 7.62 mm pixel resolution, was used to measure balance at a sampling frequency of 250 Hz. One 30-s trial of a double-leg standing balance test was performed with the participants barefooted and arms crossed over their chest with eyes opened. After a 30-s interval, another 30-s trial for single-leg standing balance was performed for each leg, similar to the double-leg standing balance test. The total center of pressure (COP) excursion was determined as the balance parameter. The average of the left and right sides was used to indicate single-leg balance ability.

### Diagnosis of HSI

HSIs were diagnosed by the team’s orthopedic physician and monitored by the team’s medical staff under the supervision of the team physician. For every injury, the date, type (e.g., strain), diagnosis (e.g., HSI), and site (e.g., left hamstring) were documented. An injury was defined as a physical complaint that requires restricted activity for at least 1 day and was only considered when it occurred during an American football practice or game [33] within one season after medical assessment.

### Statistical analysis

The statistical analyses were performed using the BellCurve for Excel (SSRI CO., Ltd., Tokyo, Japan). Parameters except for those involving the lower limbs were compared between injured and uninjured players. Parameters involving the lower limbs were compared between the injured and uninjured lower limbs of injured players and between the injured lower limbs and average for the lower limbs of uninjured players. Paired and unpaired two-tailed student’s t tests were used to assess differences in anthropometric measurements, muscle flexibility tests, muscle strength tests, balance tests, joint laxity tests. Fisher’s exact test was used to assess differences in the history of HSI. Statistical significance was set at *p* < 0.05.

According to a previous study [19], the estimated intragroup standard deviation of the H/Q was 0.08, and the estimated difference between the HSI and uninjured groups was > 0.06. This study required > 19 players with HSI to have a power of 80% and an alpha of 0.05.

## Results

HSI occurred in a total of 25 thighs from 25 players (32.1%). The incidence of HSI in this study was higher than that in previous studies, 8.7%–28.0% [7, 19]. This may be because we limited the participants to American football players playing skill positions, who have a higher risk of HSI [4, 7, 9]. None of the participants were lost to follow-up during the injury registration period.

### Anthropometric measurements

Anthropometric data are presented in Table 1. No significant differences in age, years of experience, weight, height, BMI, body muscle mass, body fat mass, and fat mass percent body, as well as history of HSI (*p* = 0.07), were observed between injured and uninjured players.

**Table 1:** Characteristics of uninjured players and those with HSI

	**Injured players (*n* = 25)**	**Uninjured players (*n* = 53)**	***p* value**
age, years	20.4 ± 0.9	20.5 ± 1.0	0.54
years of experience, years	1.3 ± 0.8	1.6 ± 1.3	0.13
weight, kg	79.0 ± 7.4	78.9 ± 6.1	0.98
height, cm	172.4 ± 4.7	173.9 ± 5.4	0.23
body mass index, kg/m^2^	26.6 ± 2.1	26.1 ± 2.1	0.43
body muscle mass, kg	60.1 ± 5.7	60.3 ± 5.2	0.72
body fat mass, kg	15.1 ± 4.6	14.7 ± 4.2	0.74
fat mass percent body, %	19.1 ± 4.9	18.7 ± 4.7	0.75
history of HSI, players	11	14	0.07

HSI: hamstring strain injury

* : significant difference (*p* < 0.05)

The data are presented as average ± standard deviation

### Muscle flexibility

The results for the muscle flexibility tests are summarized in Tables 2 and 3. No significant differences in muscle flexibilities were observed between the injured and uninjured legs of injured players (Table 2). However, injured players had significantly lower hamstring muscle flexibility compared to uninjured players (injured: 25.3 ± 9.3°, uninjured: 19.8 ± 7.7°, *p* = 0.02), although no differences in the flexibility of other muscles, namely the iliopsoas, quadriceps, gastrocnemius, and soleus, were noted (Table 3).

**Table 2:** Comparative results of the lower limb parameters between the injured and uninjured limbs of injured players

	**Injured players(*n* = 25)**		
	**Injured limb (*n* = 25)**	**Uninjured limb (*n* = 25)**	***p* value**
< muscle flexibility tests >			
iliopsoas muscle flexibility, degree	7.9 ± 3.2	7.8 ± 3.4	0.88
quadriceps muscle flexibility, degree	26.7 ± 6.7	28.0 ± 7.1	0.46
hamstring muscle flexibility, degree	25.3 ± 9.3	24.4 ± 8.3	0.50
gastrocnemius muscle flexibility, degree	9.8 ± 4.4	10.5 ± 4.1	0.17
soleus muscle flexibility, degree	17.8 ± 6.1	17.6 ± 5.5	0.86
< joint range of motion >			
hip internal rotation angle, degree	36.2 ± 8.9	35.0 ± 8.0	0.32
knee extension angle, degree	0.12 ± 5.4	1.56 ± 4.9	0.09
ankle dorsal flex angle, degree	49.5 ± 5.5	46.7 ± 4.8	0.32
< muscle strength tests >			
isometric knee extension, Nm	240.0 ± 43.7	227.1 ± 51.8	0.18
isometric knee extension / BW, Nm/kg	298.4 ± 56.3	280.8 ± 63.7	0.14
isometric knee flexion, Nm	111.6 ± 28.3	112.4 ± 29.7	0.86
isometric knee flexion / BW, Nm/kg	141.8 ± 35.0	142.1 ± 36.1	0.96
H/Q	0.47 ± 0.11	0.50 ± 0.11	0.30
isometric hip abduction, Nm	181.5 ± 32.9	185.1 ± 33.2	0.26
isometric hip abduction / BW, Nm/kg	231.4 ± 44.5	236.2 ± 45.7	0.27
< balance tests >			
COP in single-leg balance, mm	496.5 ± 153.3	489.2 ± 117.7	0.73

BW: body weight, H/Q: hamstring to quadriceps strength ratio

* : significant difference (*p* < 0.05)

The data are presented as average ± standard deviation

**Table 3:** Comparative results of the lower limb parameters between the injured limbs of injured players and the average of both lower limbs of uninjured players

	**Injured players (*n*=25)**	**Uninjured players (*n*=53)**	
	**Injured limb (*n*=25)**	**Average of limbs (*n*=53)**	***p* value**
< muscle flexibility tests >			
iliopsoas muscle flexibility, degree	7.9 ± 3.2	7.6 ± 3.1	0.65
quadriceps muscle flexibility, degree	26.7 ± 6.7	25.8 ± 4.8	0.39
hamstring muscle flexibility, degree	25.3 ± 9.3	19.8 ± 7.7	0.02*
gastrocnemius muscle flexibility, degree	9.8 ± 4.4	11.2 ± 4.6	0.20
soleus muscle flexibility, degree	17.8 ± 6.1	19.6 ± 5.5	0.21
< joint range of motion >			
hip internal rotation angle, degree	36.2 ± 8.9	37.2 ± 9.7	0.64
knee extension angle, degree	0.12 ± 5.4	0.38 ± 4.9	0.84
ankle dorsal flex angle, degree	49.5 ± 5.5	49.0 ± 6.0	0.71
< muscle strength tests >			
isometric knee extension, Nm	240.0 ± 43.7	234.6 ± 45.3	0.62
isometric knee extension / BW, Nm/kg	298.4 ± 56.3	296.5 ± 53.1	0.89
isometric knee flexion, Nm	111.6 ± 28.3	120.2 ± 21.4	0.19
isometric knee flexion / BW, Nm/kg	141.8 ± 35.0	151.8 ± 24.5	0.22
H/Q	0.47 ± 0.11	0.53 ± 0.09	0.047*
isometric hip abduction, Nm	181.5 ± 32.9	180.9 ± 35.0	0.95
isometric hip abduction / BW, Nm/kg	231.4 ± 44.5	229.1 ± 39.3	0.83
< balance tests >			
COP in single-leg balance, mm	496.5 ± 153.3	481.0 ± 112.7	0.67

BW: body weight, H/Q: hamstring to quadriceps strength ratio

* : significant difference (*p* < 0.05)

The data are presented as average ± standard deviation

### Joint range of motion

The joint range of motion is detailed in Tables 2 and 3 No significant differences in hip internal rotation, knee extension, and ankle dorsal flexion were observed between the injured and uninjured legs of injured players (Table 2) or between the injured legs of injured players and the average of uninjured players’ legs (Table 3).

### Muscle strength tests

Muscle strength data are presented in Tables 2 and 3 No significant difference in muscle strength was observed between the injured and uninjured legs of injured players (Table 2). However, although no differences in knee extension, knee flexion, and hip abduction were observed between injured and uninjured players, injured players had a significantly lower H/Q ratio than uninjured players (injured: 0.47 ± 0.11 vs. uninjured: 0.53 ± 0.09; *p* = 0.047) (Table 3).

### General joint laxity

General joint laxity scores are presented in Table 4. Injured players demonstrated significantly lower joint laxity in the elbow (injured: 1.08 ± 0.90 points, uninjured: 1.58 ± 1.07 points, *p* = 0.04) and hip (injured: 0 point, uninjured: 0.13 ± 0.34 points, *p* = 0.007). Additionally, injured players had a total score of 1.08 ± 0.90 points, which was significantly lower than that of uninjured players (1.58 ± 1.07 points, *p* = 0.04).

**Table 4:** Comparison of injured and uninjured players

	**Injured players (*n* = 25)**	**Uninjured players (*n* = 53)**	***p* value**
< joint laxity tests >			
total point / 7 points	1.08 ± 0.90	1.58 ± 1.07	0.04*
wrist	0.10 ± 0.28	0.21 ± 0.37	0.17
shoulder	0.06 ± 0.22	0.15 ± 0.27	0.12
elbow	0.02 ± 0.10	0.11 ± 0.29	0.04*
spine	0.24 ± 0.43	0.40 ± 0.49	0.16
hip	0	0.13 ± 0.34	0.007*
knee	0.5 ± 0.47	0.36 ± 0.44	0.22
ankle	0.16 ± 0.31	0.23 ± 0.37	0.42
< balance test >			
COP in double-leg balance, mm	39.8 ± 18.6	43.8 ± 20.9	0.41

COP: center of pressure

* : significant difference (*p* < 0.05)

The data are presented as average ± standard deviation

### Balance tests

Balance data are detailed in Tables 2, 3 and 4. No significant differences in COP excursion during double and single-leg balance tests were observed between the injured and uninjured players. Tables 2 and 3 also shows the results of the comparison between the injured limb and contralateral uninjured limb among injured players. No significant difference in any factors was observed between both groups.

## Discussion

The most important findings of the present prospective study were that lower hamstring flexibility, lower hamstring to quadriceps strength ratio, and lower general joint laxity may be risk factors for HSI in American football players in skilled positions. Furthermore, this is the first study on the risk factors for HSI in American football players, with a particular emphasis on skilled positions. The cohort had a high incidence of HSI.

Our findings revealed that lower hamstring flexibility was one of the significant risk factors for HSI. HSI occurs during the late swing phase of sprinting, during which the biceps femoris long head (BFLH) is elongated the most [31, 34–37] but the hamstring is in eccentric contraction. The late swing phase starts from peak knee flexion, followed by peak hip flexion, and then ends at foot strike. Higashikawa et al. [38] had shown that peak activation time of the BFLH muscle occurred in the latter half of the late swing phase, during which maximum length of the hamstring muscles was noted. Accordingly, hamstrings with lower flexibility could be relatively more elongated at the late swing phase, thereby promoting HSI. Recent studies have shown that hamstring flexibility affects the angle–torque relationship of the knee flexors [39]. Indeed, Alonso et al. [39] reported that tighter hamstrings had lower strength at long muscle lengths (shallow knee flexion angle) compared to more flexible hamstrings.

Meanwhile, van Dyk et al. [23], who assessed hamstring flexibility through passive knee extension test, similar to the current study, showed that lower hamstring flexibility was one of the risk factors for HSI in soccer players. Watsford et al. [40] also reported that lower hamstring flexibility was one of the risk factors for HSI among Australian football players. The aforementioned study assessed hamstring flexibility using the unilateral Kham test, which required players to be aligned with the lower limb position associated with the latter part of the swing phase during running, during which the hamstring is placed under high levels of eccentric tension. On the other hand, Bennell et al. [18] showed that lower hamstring flexibility was not a risk factor for HSI in Australian football players. However, they measured flexibility using the toe-touch test and concluded that the toe-touch test could not be a reliable test for screening HSI given that it did not simulate the hamstring and hip positions of the late swing phase. Thus, simulating situations that promote the occurrence of HSI may be important when evaluating the significance of muscle flexibility.

For a prevention standpoint, maintaining hamstring flexibility is considered imperative. In fact, recent reports have suggested that dynamic stretching seems to be more suitable than static stretching, given that significant reductions in maximal voluntary strength, muscle power, or evoked contractile properties have been recorded immediately after a single bout of static stretching [20].

The current study found that lower H/Q was one of the risk factors for HSI. In the swing-back motion during sprinting, the hamstring contracts eccentrically, but the antagonist muscle, namely the quadriceps femoris, contracts concentrically. This causes the hamstring to be stretched while contracting, leading to HSI [37]. Individuals with relatively weak hamstring strength and/or relatively strong quadriceps femoris strength experience more stretching of the eccentrically contracting hamstring due to concentrically contracting quadriceps femoris creating an imbalance between the hamstring and the quadriceps femoris that could be a risk factor for HSI.

Lee et al. [19] reported that soccer players with a preseason concentric H/Q below 50.5% increased had a three-fold risk for HSI. Moreover, Cameron et al. [41], who measured H/Q using relative peak torques, similar to the current study, reported that H/Q was a significant predictor of HSI in Australian football players. However, Bennel et al. [18], who measured H/Q as absolute concentric peak torque, found that H/Q was not a significant screening test for Australian football players. Although which measurement is appropriate for evaluating muscle strength still remains controversial, simulating situations causing the occurrence of HSI may be important.

Avoiding lower H/Q may be advantageous for preventing HSI. Strength training increases muscle strength but also alters muscle architecture and flexibility [42]. While Nordic hamstring eccentric strength training (NH) is an effective HSI-prevention method, the protective mechanism of this exercise has yet to be understood. Bourne et al. [43] reported that the benefits of NH are likely to be at least partly mediated by increases in BFLH fascicle length and improvements in eccentric knee flexor strength. Seymore et al. [42] suggested that NH could increase the volume and cross-sectional area of the BFLH but could not change its fascicle length or flexibility. This could indicate that NH may improve the H/Q and improve muscle flexibility, which could help prevent HSI.

This study found that lower general joint laxity was a risk factor for HSI, although many other studies have shown higher general joint laxity to be a risk factor for lower limb injuries [44, 45]. However, no studies have directly reported the association between HSI and general joint laxity. Therefore, future studies could show the reason why lower general joint laxity was a risk factor for HSI. Although general joint laxity is not modifiable, we could consider players who have lower general joint laxity to be at a higher risk of HSI.

A systematic review by Green et al. [46] revealed that the strongest risk factors for HSI were older age and history of HSI. However, the current study found no significant difference in age between injured and uninjured players. This may have been attributed to the characteristics of our cohort, which comprised only college male American football players who did not differ in age. Moreover, our findings showed that a history of HSI tended to be a risk factor for HSI (*p* = 0.07). The number of HSI history may not be sufficient to make a significant difference because most participants began playing American football after joining university, and thus the years of the experience are too short to have the history of HSI. Considering this limitation of our study, future studies should include a wider range of players.

### Limitations

This study has some limitations worth noting. First, our study included a limited number of participants. Because the preliminary power analysis indicated that 76 participants would be required and the number of participants was 78 in this study, we thought that we had enough participants. However, the post-hoc analysis showed that the number was inadequate. The post-hoc power analysis showed that the power for the H/Q was 0.30 and that for hamstring flexibility was 0.73, neither of which was large. If more participants had been included in this study, a multivariate analysis could have been performed to identify independent risk factors for HSI. Furthermore, the participants of this study were limited to colligate American football players in skill positions from only one team, the results of this study cannot be generalized to all American football players. Therefore, future large cohort studies that include players at various level categories including professional players are warranted. Second, though the hamstrings are separated into the BFLH and other muscles, this study did not assess which muscle had been injured, as well as the severity of HSI. Third, we only measured concentric isometric muscle strength. To assess the risk for HSI, muscle strength should be measured isokinetically to simulate situations in which HSI can occur [47–49]. In addition, it is recommended that hamstring muscle strength should be measured eccentrically [48]. Future studies should include the isokinetic muscle strength measurement.

## Conclusions

The current study showed that lower hamstring flexibility and lower hamstring to quadriceps strength ratio were risk factors for HSI among male college American football players placed in skill positions. However, the number of participants in this study is not large enough, and the study was limited to only one team of colligate players in skill positions, therefore the results of this study cannot be generalized to all players in American football.

## Data Availability

The datasets generated and/or analyzed during the current study are available from the corresponding author on reasonable request.

## Abbreviations

HSI: Hamstring strain injuries
H/Q: Hamstring to quadriceps strength ratio
AE: Athlete-Exposures
BW: Body weight
BMI: Body mass index
COP: Center of pressure
BFLH: Biceps femoris long head
NH: Nordic hamstring eccentric strength training
